# SIRT6 delays cellular senescence by promoting p27Kip1 ubiquitin-proteasome degradation

**DOI:** 10.18632/aging.101038

**Published:** 2016-09-16

**Authors:** Ganye Zhao, Hui Wang, Chenzhong Xu, Pan Wang, Jun Chen, Pengfeng Wang, Zhaomeng Sun, Yuanyuan Su, Zhao Wang, Limin Han, Tanjun Tong

**Affiliations:** ^1^ Peking University Research Center on Aging, Department of Biochemistry and Molecular Biology, Peking University Health Science Center, Beijing Key Laboratory of Protein Posttranslational Modifications and Cell Function, Beijing, 100191, China; ^2^ MOE Key Laboratory of Protein Sciences, Department of Pharmacology, School of Medicine, Tsinghua University, Beijing, 100084, China

**Keywords:** SIRT6, p27kip1, acetylation, ubiquitination, cellular senescence

## Abstract

Sirtuin6 (SIRT6) has been implicated as a key factor in aging and aging-related diseases. However, the role of SIRT6 in cellular senescence has not been fully understood. Here, we show that SIRT6 repressed the expression of p27^Kip1^ (p27) in cellular senescence. The expression of SIRT6 was reduced during cellular senescence, whereas enforced SIRT6 expression promoted cell proliferation and antagonized cellular senescence. In addition, we demonstrated that SIRT6 promoted p27 degradation by proteasome and SIRT6 decreased the acetylation level and protein half-life of p27. p27 acetylation increased its protein stability. Furthermore, SIRT6 directly interacted with p27. Importantly, p27 was strongly acetylated and had a prolonged protein half-life with the reduction of SIRT6 when cells were senescent, compared with those young cells. Finally, SIRT6 markedly rescued senescence induced by p27. Our findings indicate that SIRT6 decreases p27 acetylation, leading to its degradation via ubiquitin-proteasome pathway and then delays cellular senescence.

## INTRODUCTION

Sirtuins are highly conserved NAD^+^-dependent deacylases and/or mono-ADP-ribosyltransferase that have been shown to regulate lifespan in several organisms [1]. Founding member of the sirtuin family, Sir2 (silencing information regulator 2), which promotes longevity in yeast, Caenorhabditis elegans and Drosophila melanogaster, was originally discovered in Saccharomyces cerevisiae [2–5]. There are seven sirtuins in mammals, which are categorized into four groups based on their sequence homology and each family member has distinct functions and subcellular localizations [6–8]. SIRT6 is predominantly located in the nucleus [7, 11] and belongs to the class IV sirtuins, displays deacylase and ADP-ribosyltransferase activities [9, 10]. These seven proteins play key roles in a wide variety of cellular and physiological processes such as cell proliferation, differentiation, genome stability, metabolism, energy homeostasis, aging and cancer [11–15]. SIRT6-deficient mice are small and develop several acute degenerative processes that include profound lymphopenia, loss of subcutaneous fat, lordokyphosis, and severe metabolic defects at 2-3 weeks of age. These mice eventually die at about 4 weeks. These studies highlight the importance of SIRT6 in aging, metabolism and cancer for the first time [16]. Subsequent studies link SIRT6 with genomic stability, DNA repair, glucose metabolism, cancer, lipid metabolism, inflammation and heart disease [17–25].

Aging is the progressive decline in intrinsic physiological function [26]. Cellular senescence imposes permanent proliferative arrest on cells in response to variety of stressors [27]. Cellular senescence reflects organism aging and is an important contributor to aging and aging-related disease [27]. Our lab mainly focuses on the molecular mechanisms of cellular senescence [28–31]. The role of SIRT6 in cellular senescence has not been fully understood. Previous studies revealed that the p16^INK4a^ (p16)/Rb pathway, the p53/p21^Cip1^ (p21) pathway and the PTEN/p27 pathway are three key senescence-inducing pathways [32]. However, the relationship between SIRT6 and these three pathways remains to be determined.

In this study, we examined the role of SIRT6 in cellular senescence by assessing the senescent phenotypes associated with SIRT6 overexpression and small hairpin RNA-mediated SIRT6 silencing. We demonstrated that SIRT6 suppressed senescence-associated features of human embryonic lung diploid fibroblast 2BS cells by modulating p27 protein levels. SIRT6 decreased p27 at the post-transcriptional level without influencing its mRNA. We also showed that SIRT6 reduced the protein half-life of p27 through accelerating the ubiquitination of p27. In addition, SIRT6 decreased the acetylation of p27 and promoted its degradation. Moreover, SIRT6 interacted with p27 in vivo and in vitro. Furthermore, SIRT6 rescued the senescent phenotypes induced by p27. Together, our data suggest that SIRT6 suppresses cellular senescence through influencing the acetylation and ubiquitination of p27.

## RESULTS

### Expression of SIRT6 is decreased during senescence in human fibroblasts

**Figure 1:**
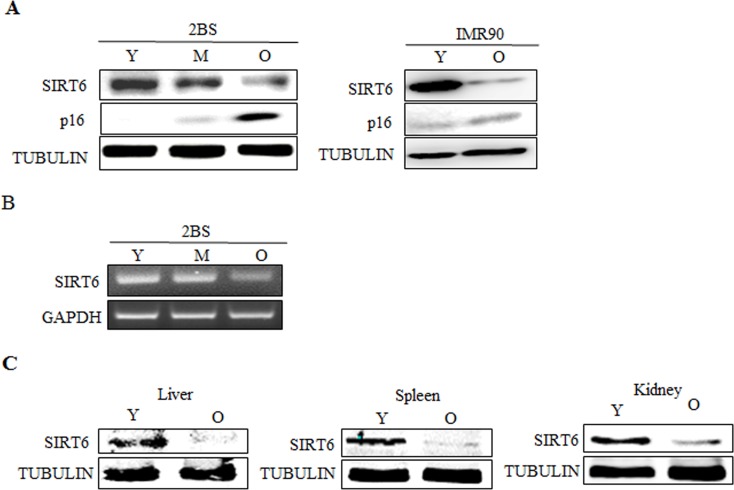
Expression patterns of SIRT6 in young and senescent cells (**A**) Left, Western blot analysis of SIRT6 expression in young (Y, ≈PD 30), middle-aged (M, ≈PD 40) and senescent (O, ≈PD 55) 2BS cells. Total protein was extracted, and immunoblotting was performed using specific antibodies against SIRT6, p16 as indicated. Tubulin served as a loading control. Right, the levels of SIRT6, p16 and TUBULIN in young (Y) and senescent (O) IMR90 cells were analysed by western blot analysis. (**B**) RT-PCR analysis of SIRT6 in young, middle-aged and senescent 2BS cells. Total mRNA was extracted and assessed by RT-PCR using specific primers. GAPDH was used as a loading control. (**C**) Protein levels of SIRT6 in indicated tissues of young (3 months) and old (18 months) BALB/c mice were determined with western blotting. Tubulin serves as loading control.

To investigate the role of SIRT6 in cellular senescence, we first examined SIRT6 expression patterns in young and senescent 2BS and IMR90 cells. Western blot analysis revealed that the expression of SIRT6 was high in young cells, but decreased significantly during cellular senescence (Figure 1A). Consistently, RT-PCR analysis revealed that mRNA levels of SIRT6 decreased in senescent cells (Figure 1B). This passage-dependent reduction suggested that SIRT6 might be involved in the process of 2BS cellular senescence. In order to examine the expression change of SIRT6 with aging in vivo, we compared its protein levels in tissues from young adult BALB/C mice (3 months of age) with those from older ones (18 months). There was a significant decrease of SIRT6 in liver, spleen and kidney of aged mice, which is comparable to results obtained from in vitro studies (Figure 1C).

### SIRT6 overexpression delays cellular senescence, whereas SIRT6 silencing results in premature senescence in human fibroblasts

**Figure 2:**
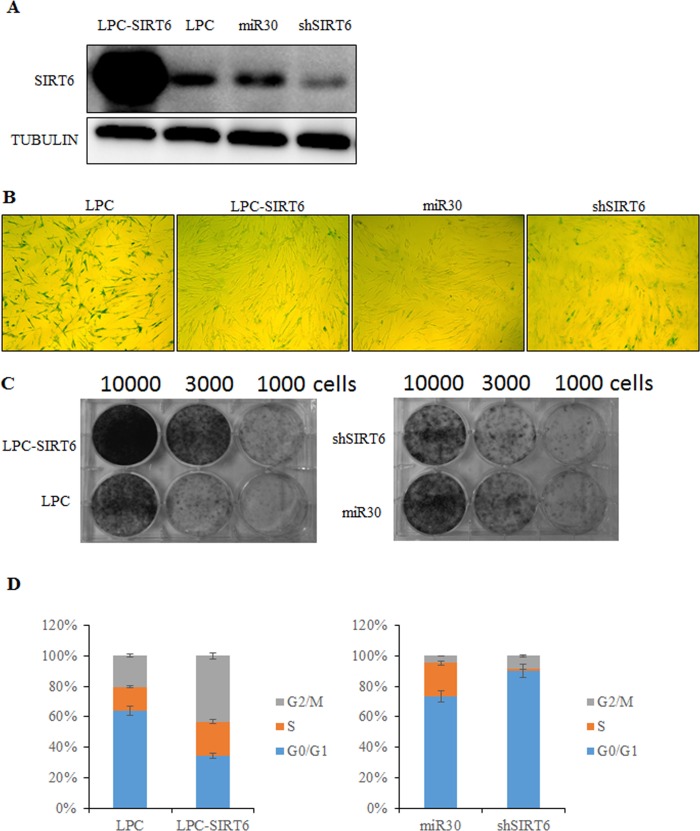
SIRT6 represses senescent-associated features in 2BS cells (**A**) Western blot analysis of SIRT6 expression levels in SIRT6-overexpression (LPC-SIRT6) and SIRT6-knockdown (shSIRT6) 2BS cells. (**B**) SIRT6-overexpression and SIRT6-knockdown 2BS cells were stained for SA-β-gal activity. (**C**) Colony formation assay was performed using SIRT6-overexpression and SIRT6-knockdown 2BS cells. (**D**) Flow cytometry analysis of SIRT6-overexpression and SIRT6-knockown 2BS cells. Values represent the means ± S.E. of triplicate points from a representative experiment (n=3), which was repeated three times.

To determine the effect of SIRT6 on cellular senescence, SIRT6 was overexpressed and silenced, respectively, with a retrovirus expression system in young 2BS cells (Figure 2A). Senescent markers for SIRT6-overexpressing and SIRT6 shRNA-infected cells were then monitored at the same time points as their corresponding control cells (Figure 2). As shown in Figure 2, SIRT6 contributed to the process of cellular senescence.

Senescence-associated β-galactosidase (SA-β-gal) staining is considered as a specific senescence marker. Most of the SIRT6-shRNA-transfected cells showed strong blue SA-β-gal staining similar to senescent cells (Figure 2B). In contrast, only scattered SA-β-gal-positive staining was found in SIRT6-transfected cells compared with the corresponding control cells (Figure 2B).

Another hallmarker of senescent cells is the ability of colony formation. We observed that 2BS cells over-expressing SIRT6 formed more colonies than control cells. However, SIRT6 shRNA-infected cells showed less colonies compared to control cells (Figure 2C).

The effects of SIRT6 on cell cycle distribution were examined by flow cytometry. 2BS cells overexpressing SIRT6 showed increased S but reduced G_1_ com-partments. It was obvious that SIRT6 shRNA-infected 2BS cells entered G_1_ cell cycle arrest (Figure 2D). Thus, SIRT6 may promote human fibroblast (2BS) proliferation by stimulating cell cycle progression.

### SIRT6 reduces protein levels of p27

**Figure 3:**
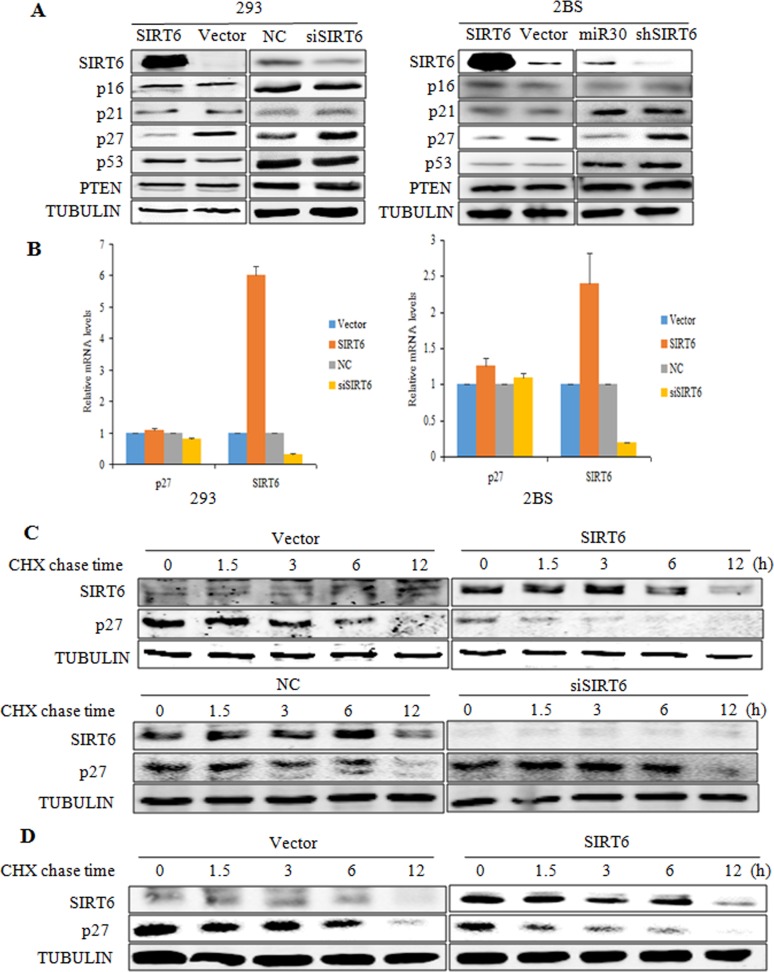
SIRT6 decreases p27 protein levels (**A**) Western blot analysis of SIRT6, p16, p21, p27, p53 and PTEN expression levels was carried out in SIRT6 overexpressing or knockdown 293 (left) and 2BS (right) cells. (**B**) Real-time PCR analysis of SIRT6, p27 was performed in cells as in A. Specific real-time PCR primers were used. (**C**) The protein half-life of p27 was evaluated in 293 cells with altered SIRT6 expression. (**D**) The protein half-life of p27 was evaluated in 2BS cells with SIRT6 overexpression.

We then examined the molecular mechanism underlying SIRT6-induced delay in cellular sense-cence. p16, p21, p27, p53 and PTEN play important roles in cellular proliferation, the influences of SIRT6 on these key factors of cellular senescence were determined. We first analyzed the expression patterns of these proteins using western blot analysis and real- time PCR. As shown in Figure 3A, the protein levels of p27 were markedly depressed in SIRT6-transfected 293 cells as compared with the vector-transfected cells, whereas the protein levels of p16, p21, p53 and PTEN were not altered. In addition, SIRT6 silencing did not alter the protein levels of p16, p21, p53 and PTEN, but markedly increased p27 protein levels. Moreover, the real-time PCR analysis demonstrated similar p27 mRNA levels in both SIRT6-overexpressing and knockdown cells, suggesting that SIRT6 might decrease the p27 protein levels but not the mRNA levels. Similar results were also observed in 2BS cells (Figure 3A and 3B).

To determine whether SIRT6 mediated the decrease of p27 protein levels post-transcriptionally, we performed a cycloheximide chase experiment in 293 cells. As shown in Figure 3C, overexpression of SIRT6 in 293 cells significantly decreased the protein half-life of p27. As expected, the protein half-life of p27 was prolonged in SIRT6 siRNA-transfected cells (Figure 3C). Comparable results were obtained in 2BS cells (Figure 3D). These data suggest that SIRT6 decreases p27 protein levels by shortening its protein half-life.

### SIRT6 promotes ubiquitination of p27

**Figure 4:**
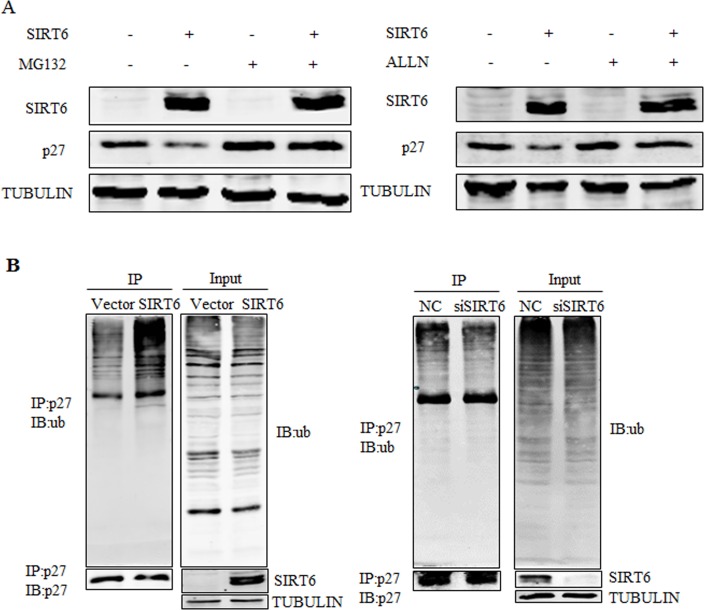
SIRT6 promotes the ubiquitination of p27 (**A**) 293 cells were transfected with SIRT6 and vector, twenty four hours after transfection, cells were treated with MG132 or acetyl-leu-leu-norleucinal (ALLN) 5 h before harvesting. Proteins were analyzed by western blot. (**B**) An in vivo ubiquitination assay was performed. 293 cells were transiently transfected with SIRT6 and vector, siRNA-SIRT6 and NC (negative control). Forty two hours later, cells were treated with MG132 for 5 h. Cells were lysed and proceeded for co-immunoprecipitation using the p27 antibody.

We subsequently examined whether SIRT6 promoted the degradation of p27. As shown in Figure 4A, SIRT6-mediated down-regulation of the p27 protein was prevented by two different proteasome inhibitors, MG132 and acetyl-Leu-Leu-norleucinal (ALLN) suggesting that SIRT6-mediated downregulation of the p27 protein might be proteasome-dependent. Then we tested the potential role of SIRT6 in the polyubiquitination of p27 in 293 cells. As shown in Figure 4B, overexpression of SIRT6 promoted the ubiquitination of p27, whereas knocking-down SIRT6 decreased the ubiquitination level of p27. These results indicate that SIRT6 induces the polyubiquitination of p27 and subsequently promotes its degradation.

### SIRT6 regulates p27 via its deacetylation activity

**Figure 5:**
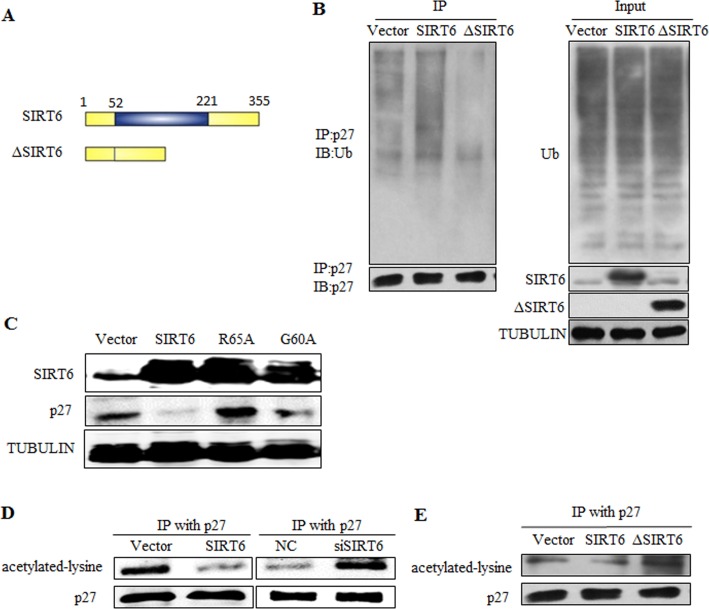
SIRT6 regulates p27 via its deacetylation activity (**A**) Schematic representation of full-length SIRT6 and its central core domain deleted mutant ΔSIRT6. (**B**) In vivo ubiquitination assay of p27 influenced by SIRT6 deleted mutant ΔSIRT6. (**C**) Overexpress SIRT6 and its mutants R65A (lack deacetylase activity) and G60A (lack mono-ADP-ribosyltransferase activity) in 293 cells, then protein levels of p27 was detected. (**D**) In vivo acetylation assay of p27 was carried out after overexpressing and knocking down SIRT6 in 293 cells. (**E**) The acetylation level of p27 was detected after overexpressing wild-type SIRT6 and its deletion ΔSIRT6.

SIRT6 has two enzymatic activities including NAD^+^-dependent deacetylation and mono-ADP-ribosylation. These two enzymatic activities both depend on its core catalytic domain. We constructed ΔSIRT6 that lacked its core domain (Figure 5A), which made its impossible to catalyze substrates. We overexpressed SIRT6 and ΔSIRT6 in 293 cells and detected the ubiquitination of p27. However, ΔSIRT6 did not alter p27 level (Figure 5B). In order to verify which enzymatic activity was important for the regulation of p27, we constructed two mutations of SIRT6: G60A lacks mono-ADP-ribosyltransferase activity and R65A lacks deacetylase activity. G60A could still decreased the protein levels of p27, whereas R65A failed to alter p27 expression, suggesting that SIRT6 plays a role in p27 degradation via its deacetylase activity (Figure 5C). Moreover, the acetylation level of p27 decreased when SIRT6 was overexpressed, whereas the acetylation level of p27 increased with SIRT6 knocking-down (Figure 5D). When SIRT6 was mutated, the acetylation level of p27 was not altered (Figure 5E).

### Acetylated-p27 is more stable

**Figure 6:**
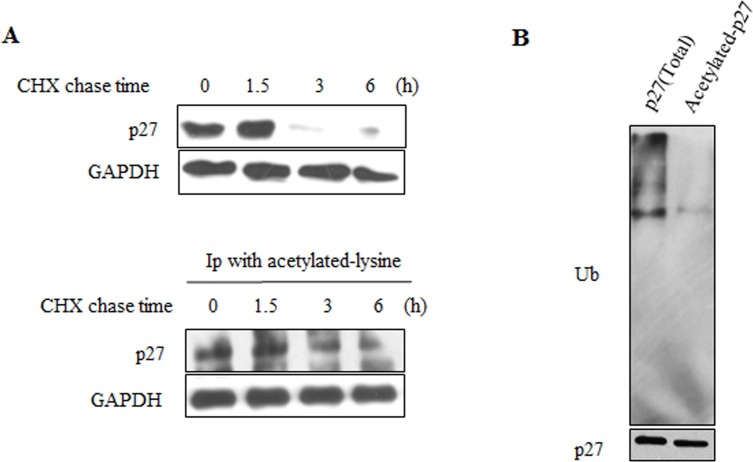
Acetylated-p27 is more stable (**A**) 293 cells were treated with CHX for different time periods, cells were then lysed and IP was performed with acetylated-lysine antibody (lower). The protein half-life of acetylated-p27 (lower) was analyzed by western blot. The protein half-life of p27 was detected as control (upper). (**B**) The ubiquitination of acetylated-p27 was detected. After treatment with MG132, 293 cells were harvested and IP was performed with acetylated-lysine antibody, then IP with p27 antibody was performed using the elution products. The second elution products were loaded on the SDS-PAGE gel for western blot to detect the ubiquitination level of acetylated-p27.

Acetylation could regulate the stability of various proteins. Acetylation of WRN could inverse its protein stability [33]. Acetylation of p53 is sufficient to block its ubiquitination and extend its half-life [34]. Unlike p53 and WRN, acetylation of cyclin A by PCAF promotes its ubiquitination [35]. As SIRT6 affects both acetylation and ubiquitination level of p27, the acetylation of p27 may affect its ubiquitination. The half-life of acetylated-p27 (Figure 6A, lower) was longer than that of total p27 protein following cycloheximide (CHX) treatment (Figure 6A, upper). As shown in Figure 6B, high levels of ubiquitinated p27 proteins were present in the total p27 protein prepared from 293 cells. In contrast, the ubiquitinated p27 was almost undetectable in the acetylated form of p27 protein (Figure 6B). These results demonstrate that acetylation of p27 prevents its ubiquitination and stabilizes the p27 protein.

### SIRT6 interacts with p27 in vivo and in vitro

**Figure 7:**
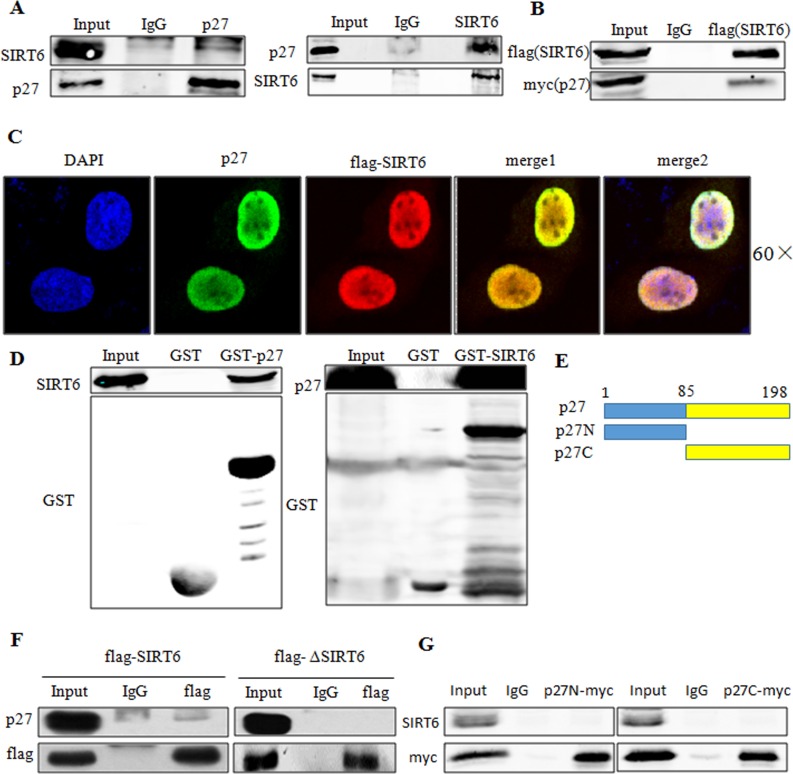
SIRT6 interacts with p27 in vivo and in vitro (**A**) Co-IP of endogenous SIRT6 and p27 was performed in 293 cells. (**B**) Co-IP of exogenous SIRT6 (flag-SIRT6) and p27 (myc-p27) was performed using flag antibody in 293 cells. (**C**) 293 cells were transfected with flag-SIRT6 and p27, then immunofluorescence assay was carried out using flag and p27 antibodies to investigate the co-localization of these two proteins. (**D**) GST-pulldown assay using in vitro transcribed and translated SIRT6 or p27 and purified GST-p27 or GST-SIRT6 from E.coli BL21 cells. Blots were evaluated with SIRT6, p27 and GST antibodies. (**E**) Schematic representation of full-length p27 and its deletions. (F) 293 cells were transfected with flag-SIRT6 and flag-ΔSIRT6. Cell lysates were then used for co-IP with the flag antibody. Blots were evaluated with flag and p27 antibody. (**G**). 293 cells were transfected with flag-SIRT6 and myc-p27N or myc-p27C, co-IP was then performed using anti-myc antibody. Blots were evaluated with myc and SIRT6 antibodies.

As SIRT6 can decrease the acetylation of p27, we next sought to determine whether this downregulation was depended on a direct interaction between SIRT6 and p27. As shown in Figure 7A, p27 could interact with SIRT6 in vivo. In addition, Co-transfection of flag-SIRT6 and myc-p27 in 293 cells revealed that exogenous SIRT6 could also interact with exogenous p27 (Figure 7B). Colocalization experiments showed that both SIRT6 and p27 mainly colocalized in the nucleus (Figure 7C). To further confirm the interaction of p27 and SIRT6, we performed a GST pull down assay using in vitro transcribed and translated p27 or SIRT6 and purified GST-SIRT6 or GST-p27. As shown in Figure 7D, SIRT6 displayed a strong association with p27 in vitro.

To determine the domain of SIRT6 required to interact with p27, we overexpressed full-length and core catalytic domain deleted (ΔSIRT6) constructs of SIRT6 in 293 cells. As shown in Figure 7F, ΔSIRT6 had no interaction with p27, suggesting that the core catalytic domain was required for the interaction of SIRT6 with p27. To identify the domain of p27 required for its interaction with SIRT6, we constructed two deletions of p27 as described in Figure 7E. Both p27N and p27C deletions disrupted the interaction between SIRT6 and p27 (Figure 7G).

### SIRT6 expression level inversely correlates with p27 acetylation level and its protein half-life during cellular senescence

**Figure 8:**
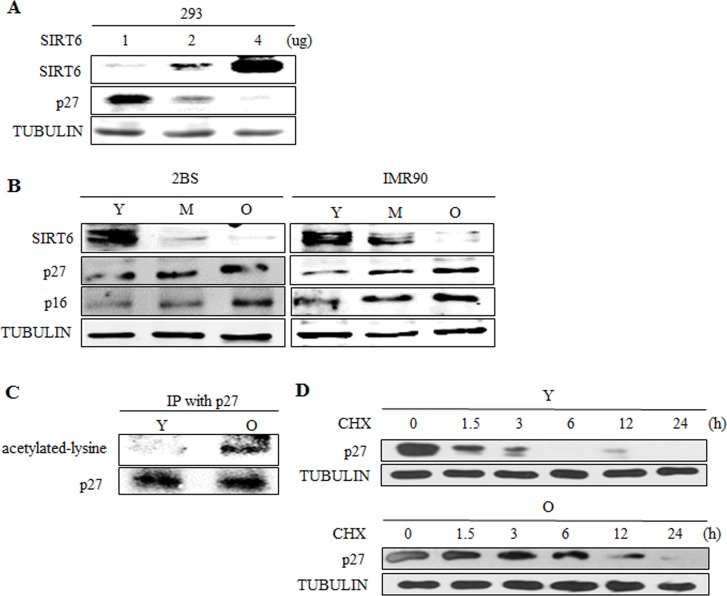
SIRT6 expression correlates with p27 acetylation level and protein half-life during cellular senescence (**A**) Different amounts of SIRT6 were expressed in 293 cells. 48 h after transfection, SIRT6 and p27 were analysed by western blot. (**B**) Western blot analysis of SIRT6, p16 and p27 expression in young (Y, ≈PD 30), middle-aged (M, ≈PD 40) and senescent (O, ≈PD 55) 2BS and IMR90 cells. (**C**) The acetylation level of p27 was detected in young (Y, ≈PD 30) and senescent (O, ≈PD 55) 2BS cells. (**D**) The protein half-life of p27 during cellular senescence in 2BS was detected.

Given of the importance of SIRT6 in cellular senescence and the role of SIRT6 in the acetylation of p27, we further evaluated the expression patterns of SIRT6 and the acetylation level of p27 in young and senescent 2BS cells. SIRT6 regulated p27 in a dose-dependent way in 293 cells (Figure 8A). The expression of SIRT6 was inversely correlated with the protein levels of p27 during the senescence of 2BS and IMR90 cells (Figure 8B). The acetylation level of p27 and the protein half-life of p27 during cellular senescence were detected. Moreover, the acetylation level of p27 increased with cellular senescence (Figure 8C) and the protein half-life of p27 was prolonged during cellular senescence (Figure 8D). As a decline in SIRT6 expression was accompanied by an increasing level of p27 acetylation and extended protein half-life of p27 during cellular senescence, it is conceivable that a rise in the level of p27 during cellular senescence may be in part attributed to the downregulation of SIRT6.

### SIRT6 inhibits cellular senescence induced by p27

**Figure 9:**
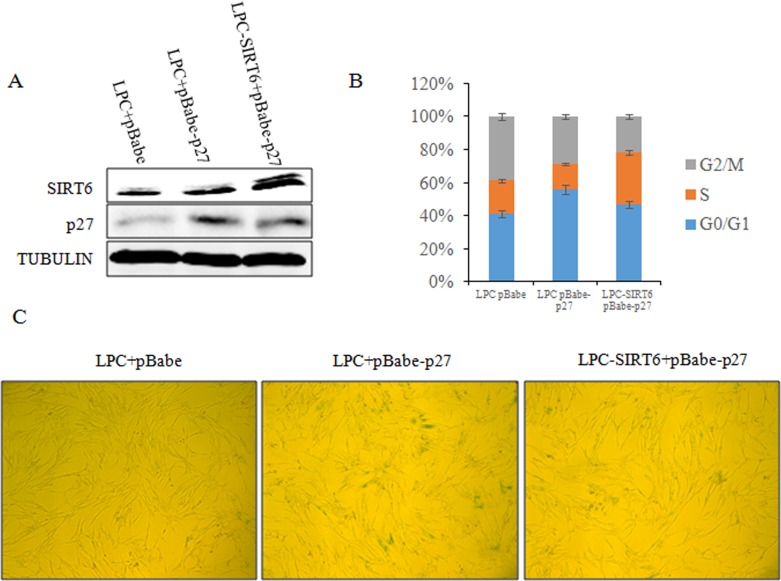
SIRT6 represses cellular senescence induced by p27 (**A**) 2BS cells were stably co-transfected with LPC vector and pBabe vector, LPC vector and pBabe-p27 or LPC-SIRT6 and pBabe-p27 plasmids. The expression of SIRT6 and p27 protein levels in the stable transformants were analyzed by western blot. (**B**) Cell cycle of cells from A was detected. Values represent the means ± S.E. of triplicate points from a representative experiments (n=3), which was repeated three times. (**C**) Cells from A were stained for SA-β-gal activity.

Given the critical role of p27 in SIRT6-mediated cellular senescence, we investigated whether SIRT6 delays the cellular senescence induced by p27. As shown in Figure 9, 2BS cells co-expressing pLPC, pBabe-p27 exhibited G_1_ arrest and higher SA-β-gal activity. Concurrently, the senescent markers of SIRT6-p27 appeared to be comparable with that of pLPC-pBabe control cells (Figure 9B and 9C). These findings strongly suggest that SIRT6 inhibited p27-induced senescence. Thus, the decrease of p27 mediated by SIRT6 is required for the restriction of cellular senescence.

## DISCUSSION

In the present study, we demonstrated that SIRT6 could delay cellular senescence by targeting p27 for ubiquitin-mediated proteasome degradation. Consistent with our findings, other groups also reported that SIRT6 diminishes with cellular senescence of a primary fore-skin fibroblast strain (HCA2) [36] and SIRT6 knockdown in WI-38 human fibroblasts leads to a strikingly shortened replicative lifespan and increased levels of SA-β-gal staining [17]. In addition, SIRT6 inhibits senescence of human umbilical vein endothelial cells (HUVEC) [37] or TGF-β induced senescence of human epithelial cells [38]. These findings indicate that SIRT6 restrains cellular senescence. However, Overexpression of SIRT6 has been shown not to prolong cellular replicative lifespan in normal human fibroblasts (NHF and WI-38) or prostate epithelial cells (PrEC). In addition, both SIRT6 mRNA and protein levels do not cause significant changes in NHF and WI-38 cells [7].

SIRT6 regulates cellular senescence through promoting DNA damage repair and suppressing genomic instability [16, 17, 24, 36]. Moreover, SIRT6 also attenuates NF-κB signalling via H3K9 deacetylation at chromatin to restrain cellular senescence [39, 40], although another group finds that SIRT6 does not influence NF-κB responses [41]. In addition, silencing of SIRT6 in HUVEC activates the p21 pathway [37], whereas overexpression of SIRT6 inhibits TGF-β-induced senescence of epithelial cells via proteasomal degradation of p21 [38]. However, SIRT6 inhibition in chondrocytes leads to p21 downregulation due to the time of the extraction of protein [42], as p21 is up-regulated during the early stage of senescence and decreases once senescence was achieved [43]. Depletion of SIRT6 leads to p16 upregulation in chondrocytes [42] and bone marrow mesenchymal stem cell (BM-MSC) [44]. Overexpression of SIRT6 decreases p16 protein levels in chondrocytes [45]. In contrast, p16 levels are undetectable when silencing SIRT6 in HUVEC cells [37]. In our results, SIRT6 has no obvious influence on p16 and p21 expression in 293 and 2BS cells (Figure 3A). Cell type differences may underlie the divergent phenotypes.

Among all the key proteins in the p21, p16 and p27 signaling pathways, only p27 protein level was significantly changed following overexpressing and knocking down SIRT6 (Figure 3A). As the upstream of p27 [32], PTEN was not required for the regulation of p27 by SIRT6 (Figure 3A). We demonstrated that SIRT6 shortened the protein half-life of p27 and increased the ubiquitination of p27 which depended on its core catalytic domain (Figure 3-5). The regulation of p27 by SIRT6 needed its deacetylation activity (Figure 5C). SIRT6 decreased the acetylation level of p27 (Figure 5D). These observations suggest that the acetylation level of p27 determines the stability of p27, which is supported by the findings that the protein half-life of acetylated-p27 was longer and that there was a low ubiquitination level of acetylated-p27 compared with that of p27 (Figure 6). In addition, SIRT6 interacted with p27 through its core catalytic domain (Figure 7). Moreover, the acetylation level of p27 increased and the protein half-life of p27 prolonged during cellular senescence with SIRT6 decreasing (Figure 8). Finally, rescued experiments further demonstrated that SIRT6 delayed cellular senescence induced by p27 (Figure 9).

It has been reported that PCAF acetylates p27 at lysine 100 (K100) and induces the degradation of p27 via proteasome [46], which is contradictory to our results. Acetylation can regulate the stability of multiple proteins by influencing its ubiquitination in different ways. Acetylation of WRN, p53 or tau is sufficient to block its ubiquitination and extends its protein half-life [33, 34, 47]. Unlike p53 and WRN, acetylation of cyclin A or E2F-1 promotes its ubiquitination [35, 48]. However, another group finds that sirtuin-mediated deacetylation pathway stabilizes WRN protein [49], which is at odds with the conclusion above. Coincidentally, Yuan et al. finds that SIRT1 interacts with c-MYC and deacetylases c-MYC at K323 to promote the degradation of c-MYC [50], whereas Menssen et al. finds that SIRT1 stabilizes c-MYC and mutation of K323 in c-MYC does not affect c-MYC stability [51]. Those discrepancies above likely arise from the different experimental approaches. The acetylated sites of p27 could also be different, which may lead to opposite observations.

Zhu et al. finds that SIRT1 reduces the half-life of p27 and accelerate its degradation. However, they did not detect its acetylation level. Whether SIRT1 could influence p27 through regulating its acetylation level still needs more evidences [52]. SIRT1, FOXO3a and NRF1 could form a complex to promote the transcription of SIRT6 [53], it would be possible that SIRT1 could inhibit p27 through SIRT6. In addition, SIRT2 induces Skp2 deacetylation and subsequent degradation, then promoting p27 protein level in non-small cell lung cancer [54]. SIRT2 can also elevate the expression of p27 through regulating FOXO3a in response to oxidative stress and caloric restriction [55]. It has not been determined whether SIRT2 can influence the acetylation level of p27. Moreover, the relationship between SIRT6 and SIRT2 has not been investigated. Furthermore, it is not clear how sirtuins are regulated by p27.

**Figure 10:**
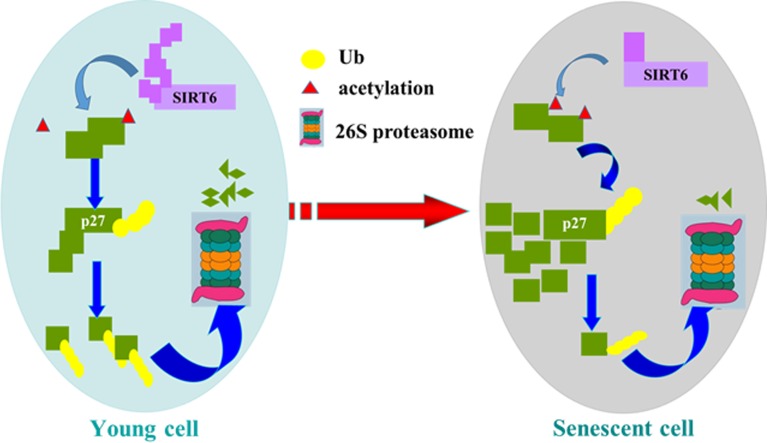
Model of SIRT6 regulating p27 during cellular senescence SIRT6 deacetylates p27, which promotes the ubiquitination of p27 and increases the degradation of p27 by proteasome. Then SIRT6 can repress cellular senescence.

In summary, our study suggest a new molecular mechanism for SIRT6 delaying cellular senescence. When cells are young, SIRT6 decreases the acetylation level of p27, which promotes p27 degradation by proteasome. The acetylation level of p27 increases with the decline of SIRT6, which improves the stability of p27 and induces cellular senescence (Figure 10). Our findings may provide a new way to resist cellular senescence and to delay aging related diseases.

## MATERIALS AND METHODS

### Cell culture and animals

2BS cells (National Institute of Biological Products, Beijing, China) and IMR90 cells (kindly provided by Masashi Narita, Cancer Research U.K., Cambridge Research Institute) are two kinds of human embryonic lung diploid fibroblasts. IMR90 cells and 2BS cells are considered to be young at 30 population doublings (PD) or below and to be senescent (O) at PD 55 or above. HEK293 cells were from our lab, and phoenix packaging cells were gifts from Dr. M. Narita. All cells were cultured in DMEM (Macgene) supplemented with 10% fetal bovine serum (Hyclone) at 37°C in 5% CO_2_. Cultures at 80% confluence were detached from the dish with 0.25% trypsin (Macgene) and then split at a ratio of 1:2. Young (Y, 3-4 months) and aged (O, 18-22 months) BALB/c mice were maintained on a 12 h light/dark cycle with food and water available. All animal studies were performed in accordance with the guidelines set forth by the Peking University Animal Ethics Committee.

### Plasmids and transfection

Full-length SIRT6 was cloned into pLPC-puro, p3×FLAG-CMV, and pGEX-4T-1. The construct ΔSIRT6 (core domain deleted) was cloned into p3×FLAG-CMV. The SIRT6 mutants R65A (lack deacetylase activity) and G60A (lack mono-ADP-ribosyltransferase activity) were generated by site-specific mutagenesis method (TransGen). All constructs are expressed fusion proteins with FLAG. p27 was placed into pBabe-neo, pcDNA3.1-Myc-His, and pGEX-4T-2 as described [30]. All clones were confirmed by DNA sequencing. p27N and p27C were cloned into pcDNA3.1-Myc-His.

The shRNA was designed according to the pMSCV instruction manual (Clontech). The double strand RNAs were synthesized by Shanghai Generay Biotechnology Co., Ltd and subsequently inserted into the EcoRI and XhoI sites of the pMCV-puro-miR30 vector. The human SIRT6-specific shRNA target sequences used was: miR30-SIRT6: 5′-AAGAATGTGCCAAGTGTAAGA-3′, which targets human SIRT6 416-436 bp [17].

For transient transfection, 293 cells at 70–80% confluence were transfected with Calcium Phosphate Cell Transfection Kit (Macgene) following the manufacturer's instructions.

For 2BS transfection, retroviral infection was used [56]. After phoenix cells reached 60–70% confluence, the retroviral plasmids were transfected with Calcium Phosphate Cell Transfection Kit according to the manufacturer's instructions. The retrovirus supernatants were collected 48 h after transfection and then filtered. 2BS cells were infected with retrovirus in the presence of 8 μg/ml Polybrene (Invitrogen). Pools of stable transformants were obtained by sustained selection with 0.5 μg/ml puromycin (Invitrogen), starting 1 day after infection.

### siRNA transfection

To transiently silence SIRT6, siRNA targeting SIRT6 was synthesized (GenePharma) and transfected with Lipofectamine™ RNAiMAX (Invitrogen) following the manufacturer's instructions. Cells were collected 72 h after transfection for further analysis.

### Western blot analysis

Cells were washed with PBS, collected, and lysed on ice for 30 min with modified Radioimmune Precipitation Assay (RIPA) Buffer (Applygen) containing a protease inhibitor cocktail (Amresco). Cell lysates were then centrifuged for 15 min at 12,000 × rpm and 4°C. The supernatant was collected, and the protein concentration was measured using the BCA Protein Assay Reagent (Pierce). Total protein (30-80 μg) was subjected to 10–15% sodium dodecyl sulfate–polyacrylamide gel electrophoresis (SDS–PAGE) and was transferred to nitrocellulose membranes (Millipore). After blocking in 5% non-fat dry milk in TBST (10 mM Tris-Cl, pH 7.5, 150 mM NaCl, 0.05% Tween20), the membranes were incubated with primary antibodies overnight at 4°C. After washing in TBST buffer, membranes were incubated for 1 h in the dark with the appropriate IRDye TM 800-conjugated secondary antibodies (Thermo) in TBST/5% non-fat milk. Signals were detected on an Odyssey Infrared Imager (LI-COR Bioscience) after washing.

The following antibodies were used for the Western blot analysis: anti-SIRT6 (Abcam, CST), anti-Multi Ubiquitin (MBL), anti-FLAG (Sigma), anti-Myc (MBL), anti-p27 (MBL, Santa), anti-GAPDH (Tianjin Sungene Biotech Co., Ltd.), anti-β-TUBULIN (Santa), anti-p21 (MBL), anti-p53 (Santa), anti-p16 (Santa), anti-PTEN (Santa), anti-acetylated lysine (CST).

### Reverse transcription-PCR

Total RNA was isolated from cells using a RNeasy Mini kit (Qiagen) according to the manufacturer's instructions. First-strand cDNA was synthesized using TransScript First-Strand cDNA Synthesis SuperMix (TransGen). For the RT-PCR analysis of SIRT6 and GAPDH expression, specific primers were used: CCCACGGAGTCTGGACCAT and CTCTGCCAGTTTGTCCCTG for SIRT6 mRNA; CGAGTCAACGGATTTGGTGGTAT and AGCCTTCTCCATGGTGAAGAC for GAPDH mRNA. PCR was performed and expression level of mRNA was assessed by staining gels with GoodView^TM^ (sbsbio). For the Real-time PCR analysis of SIRT6, p27 and actin expression, specific primers were used. The primer sequences were as follows: GCTGACCTCCGCATCCAT and AGCCCCAGGTGCTTCATG for SIRT6 mRNA, GGCTCCGGCTAACTCTGA and TCTTCTGTTCTGTTGGCTCTTT for p27 mRNA, and AGCGAGCATCCCCCAAAGTT and GGGCACGAAGGCT CATCATT for actin mRNA. Real-time PCR was performed using the SYBR^®^ Select Master Mix (Applied biosystems).

### Senescence-associated-β-gal staining assay

SA-β-gal staining was conducted using senescence associated β-gal detection kit (GenMed) according to the manufacturer's instructions. Brief introduction: cells were washed twice, fixed for 5min at room temperature, and washed twice with wash buffer. The cells were then incubated in a freshly prepared SA-β-gal staining solution at 37°C without CO_2_ for indicated time.

### Immunofluorescence

Cells were cultured on glass coverslips and transfected with indicated plasmids. Cells were fixed using 4% paraformaldehyde for 10min, then permeabilized with 0.5% Triton X-100 in PBS for 10 min and incubated in blocking solution for 2 h at room temperature. Coverslips were incubated for 1 h with indicated antibodies. Cells were washed with PBS before incubation with indicated secondary antibodies. After washing with PBS, the DNA was stained with DAPI. Coverslips were mounted in a 90% glycerol solution. Coverslips were examined using a Leica confocal TCS SP2 microscope.

### Cell cycle analysis

When cells reached 70–80% confluence, they were washed with PBS, detached with 0.25% trypsin, and fixed with 75% ethanol overnight at 4°C. Cells were washed twice with cold PBS, then treated with 50 μg/ml RNase A (Sigma) at 37°C for 30 min, after that, cells were resuspended in 0.5 ml of PBS and stained with propidium iodide in the dark for 30 min. Fluorescence was measured with a FACScan flow cytometry system (BD Biosciences).

### Co-immunoprecipitation assays

Cells for immunoprecipitation assay were lysed in immunoprecipitation lysis buffer (25 mM Tris-HCl pH 7.4, 150 mM NaCl, 1% Nonidet P-40, 1 mM EDTA, 5% glycerol) containing Protease Inhibitor Mixture (Amresco). The whole cell lysates obtained by centrifugation were incubated with specified antibodies overnight at 4°C with constant rotation. Then appropriate amount of PureProteome Protein A and Magnetic Beads (Millipore) were added to the sample and incubated at 4°C with constant rotation for 1 h. The immunocomplexes were then washed with immunoprecipitation lysis buffer three times, boiled in SDS sample buffer, and subjected to SDS-PAGE followed by Western blot analysis.

### GST pulldown assays

Expression of fusion proteins were induced with isopropyl β-D-1-thiogalactopyranoside in Escherichia coli strain BL21, then purified with glutathione-conjugated sepharose beads (GE Healthcare) following the manufacturer's instructions. The other proteins were transcribed and translated in vitro using the TNT T7 Quick Coupled Transcription/Translation System (Promega). The purified bacterial recombinant protein immobilized on glutathione-conjugated sepharose beads was incubated with in vitro transcribed/translated proteins for 4 h on a rotator at 4°C. After extensive washing with lysis buffer, the bound proteins were subjected to Western blot analysis.

### Colony formation assay

1 × 10^3^, 3 ×10^3^, and 1 ×10^4^ cells were cultured in six-well plate. Several days later, cells were fixed in 4% formaldehyde at room temperature for 30 min and washed twice with 1 × PBS, then stained with crystal violet for 30 min and washed with 1 × PBS twice.

### In vivo ubiquitination assay

Cells were treated with 20 μM MG132 for 5 h before harvesting, and the cells were lysed in lysis buffer. Then the cell extracts were immunoprecipitated with p27 antibody, The immunoprecipitates were subsequently resolved on SDS-PAGE gels and the ubiquitination level of p27 was analyzed by Western blot with multi-ubiquitination antibody.

### In vivo acetylation assay

Cell extracts were immunoprecipitated with p27 anti-body. The immunoprecipitates were subsequently resolved on SDS-PAGE gels and the acetylation level of p27 was analyzed by Western blot with acetylated-lysine antibody. To avoid p27 deacetylation during the preparation, 1 μM TSA (sigma) and 5mM nicotinamide (sigma) were added in each step.

For preparing the total p27 protein, the cell extracts were immunoprecipitated with a p27 antibody. For preparing the acetylated p27 protein, the cell extracts were first IP by the acetylated-lysine antibody and then immunoprecipitated by the p27 antibody.

### Protein half-life assay

Cycloheximide (CHX, Sigma) was dissolved in DMSO and added to cells at 100 μg/ml for the indicated time periods. The whole cell lysates were made, and protein concentration was determined. Subsequently, appropriate amount of total protein from each sample was analyzed by western blot.
